# New Modified UPLC/Tandem Mass Spectrometry Method for Determination of Risperidone and Its Active Metabolite 9-Hydroxyrisperidone in Plasma: Application to Dose-Dependent Pharmacokinetic Study in Sprague-Dawley Rats

**DOI:** 10.1155/2017/1271383

**Published:** 2017-05-03

**Authors:** Essam Ezzeldin, Marwa Tammam, Nisreen F. Abo Talib

**Affiliations:** ^1^Pharmaceutical Chemistry Department and Drug Bioavailability Laboratory, College of Pharmacy, King Saud University, P.O. Box 2457, Riyadh 11451, Saudi Arabia; ^2^Drug Bioavailability Center, National Organization for Drug Control and Research, P.O. Box 29, Cairo, Egypt

## Abstract

Sensitive and specific liquid-chromatography tandem mass spectrometry (UPLC-MS/MS) assay has been developed and validated for simultaneous quantification of risperidone (RIS) and its active metabolite 9-hydroxyrisperidone (9-OH-RIS) in rat plasma using olanzapine (OLA) as internal standard (IS). Pharmacokinetics of risperidone and its active metabolite 9-hydroxyrisperidone was compared across different doses (0.3, 1.0, and 6.0 mg/kg). Serial blood sample was collected over a time of 48 hours and analyzed for risperidone and its active metabolite 9-hydroxyrisperidone. The pharmacokinetics parameters including *C*_max_, *t*_max_, and AUC were determined for risperidone and its active ingredient. The method was linear in the concentration range of 0.2–500 ng/mL for risperidone and 9-OH-risperidone, with coefficients of determination greater than 0.998 and lower limit of quantitation of 0.2 ng/mL. Blood levels of risperidone and its active metabolite were roughly dose-proportional. The method developed herein is simple and rapid and was successfully applied for dose-dependent pharmacokinetic study.

## 1. Introduction

**Figure 1:**
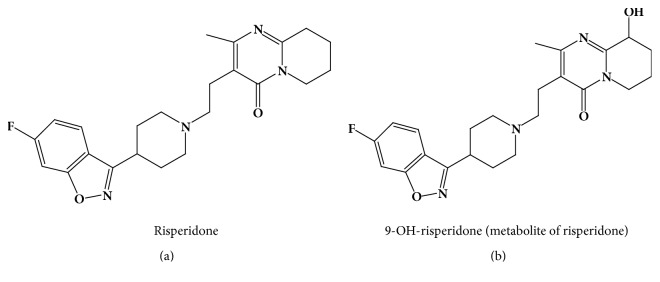
Structure of risperidone (a) and 9-OH-risperidone (b).

RIS is (3-[2-[4-(6-fluoro-1,2-benzisoxazol-3-yl)-1-piperidinyl]ethyl]-6,7,8,9-tetrahydro-2-methyl 4H-pyrido[1,2-a]pyrimidin-4-one) (Figure 1(a)). It is an atypical antipsychotic drug used in the treatment of mental and mood disorders. It is helping to restore the certain natural substances balance in the brain [1]. Combined drugs containing risperidone may also be used in depression treatment [2]. Risperidone maximum concentration (*C*_max_) occurs during two hours following oral administration. It is metabolized mainly by CYP450-2D6 (CYP2D6), through hydroxylation giving 9-OH-risperidone (Figure 1(b)), which have pharmacological effects like parent drug, risperidone [3–5]. Elimination half-life is variable in different patients according to the genetic polymorphism, and it has lasted a long time in poor metabolite individuals [6].

Previous publications have reported methods for determination of risperidone in a biological fluid using high-performance liquid chromatography (HPLC) methods with ultraviolet detection [7–15], electrochemical detection [16, 17], LC-MS [18–21], and also LC-MS/MS methods [22–25]. However, due to lack of sensitivity, the application of LC-UV on pharmacokinetic study of risperidone is disturbed. The main disadvantage of the LC-UV method is needed of large volume of samples and a long analysis time which restricted its application. The aim of the present work is the development and validation of an analytical method using UPLC-MS/MS for the determination of RIS and its active metabolite 9-OH-RIS in small volumes of plasma.

The method compromises a simple condition for chromatographic separation. Moreover, preparation and extraction process were done in one step. The method also has enough sensitive, high-throughput method and was successfully applied to the dose-dependent pharmacokinetic study of risperidone in rats.

## 2. Experimental

### 2.1. Materials

Risperidone (99.6% potency) and 9-hydroxyrisperidone (99.5% potency) standard materials were purchased from Janssen Cilag, Italy, and olanzapine (OLA) was kindly supplied from Sigma-Aldrich Co. (UK). Formic acid and ammonium acetate were purchased from Romil Chemicals, England. HPLC grade acetonitrile and methanol were purchased from Alpha Chemicals, Egypt. Deionized water and membrane filter (0.45 *μ*m Whatman) were used in the mobile phase filtration.

### 2.2. Chromatographic Conditions and UPLC-MS/MS Instrumentation

“ACQUITY™ UPLC system (Waters Corp., Milford, MA, USA)” was used to perform the chromatographic separation. “Acquity UPLC BEH™” C_18_ column (50 × 2.1 mm, i.d., 1.7 *μ*m) “(Waters, USA)” was used in the separation. Column was kept at 40°C. A mobile phase consists of acetonitrile containing 0.1% formic acid and aqueous 5 mM ammonium acetate buffer containing 0.1% formic acid (80 : 20, v/v) at a flow rate 0.5 mL/min with a run time lasted only two minutes. Five *μ*L was injected in partial loop mode. The temperature of the autosampler was maintained at 10°C. The ESI source was operated in positive ionization mode. Quantification was performed using multiple reaction monitoring (MRM) of the transitions of* m/z* 411.3 > 191.31, 426.58 > 207.29, and 312.70 > 256.29 which were used to quantify risperidone, 9-OH-risperidone, and IS, respectively. Mass spectrometry instrument was adjusted as mentioned in Table 1. All peak areas were obtained using QuanLynx software (version 4.1).

### 2.3. Standards Calibration Curve and Quality Control

Standard stock solutions RIS, 9-OH-RIS, and OLA were prepared using methanol as dissolving agent to give a final concentration of 100.0 *μ*g/mL. The prepared solutions were preserved in the refrigerator for subsequent use during two weeks from the date of preparation. To construct plasma calibration standards, appropriate amounts of diluted stock solutions of RIS, and 9-OH-RIS in methanol were added to blank plasma to yield a final concentration of 0.2, 0.5, 1.0, 5.0, 10.0, 30.0, 100.0, and 500.0 ng/mL for both drugs. Four different concentrations of QC plasma samples were prepared in similar procedures as calibration standards and they are treated as LLQC (0.2 ng/mL), LQC (2.0 ng/mL), MQC (20.0 ng/mL), and HQC (400 ng/mL). Plasma sample of calibration standard and QC samples were kept at −80°C until used for validation of bioanalytical method. Working solution of internal standard OLA (12 *μ*g/mL) was prepared in methanol and kept at 4°C.

### 2.4. Sample Preparation

Plasma samples stored at −80°C were thawed, left for one hour at room temperature, and shaken by vortex for 30 s before extraction processes. 10 *μ*L (12 *μ*g/mL) of IS was added to 100 *μ*L of spiked calibration standards, QCs, and unknown plasma samples (except blank sample). The samples were mixed by vortex for about 30 s and 300 *μ*L of acetonitrile added to it. The samples were again mixed gently by vortex for 1.5 min. After centrifugation at 4000 rpm and 4°C, 150 *μ*L of supernatant was transferred to vials, and 5 *μ*L volumes of the sample were injected to the analysis by UPLC-MS/MS.

### 2.5. Method Validation

According to guidelines set by the “United States Food and Drug Administration” [26], a full method validation was performed for the analysis of RIS and its metabolite. The validation of this method used was performed in plasma including all parameters required by the guideline.

#### 2.5.1. Selectivity and Specificity

Method selectivity for determination of RIS and 9-OH-RIS was assessed in blank plasma. Among the analyzed plasma batches, no or minimal interference at the retention time of analytes and the selected internal standard were observed. They were processed and analyzed using the proposed extraction protocol and spiked with standards RIS, 9-OH-RIS at LLOQ (0.2 ng/mL), and IS at 1200.0 ng/mL.

#### 2.5.2. Linearity and Standard Curve

Method linearity was determined by spiking various known concentrations of RIS and 9-OH-RIS to yield different concentrations of 0.2, 0.5, 1.0, 5.0, 10.0, 30.0, 100.0, and 500.0 ng/mL. Concentrations were plotted against the peak area ratios of each of risperidone and its metabolite to internal standard. The best-fit line was obtained by linear regression analysis of the resultant curve. Regression coefficient and linearity are calculated according to the equation (*y* = *mx* + *c*). The calibration curve requirement was set at a correlation coefficient (*r*^2^) which should be equal to or better than 0.997. The analyte peak of LLOQ sample should be identifiable, discrete, and reproducible with accuracy within ±20% and a precision ≤ 20%. The deviation of standards other than LLOQ from the nominal concentration should not be more than ±15.0%.

#### 2.5.3. Precision and Accuracy

Accuracy was determined by analysis of three quality control samples, low (2.0 ng/mL), medium (20.0 ng/mL), and high (400.0 ng/mL) quality control samples. Accuracy was measured by the analysis, at least six determinations per concentration. Acceptance value of accuracy is 15% within the actual sample. For precision the QCs were assayed six times in three separate essays on separate days. For the precision, coefficient of variation (CV%) should not exceed 15%.

#### 2.5.4. Recovery

Recovery is the ratio between detector responses from the extracted drug from spiked plasma to that response obtained for the concentration of pure authentic samples. Recovery experiments for analysis of RIS and 9-OH-RIS at 3 QC levels were performed by comparing the analytical results for extracted samples at three concentrations (2.0, 20.0, and 400.0 ng/mL).

#### 2.5.5. Stability

For stability determination samples were prepared from freshly prepared stock solutions. Stability evaluation includes the stability during sample collection and handling, after short-term storage, and after going through freeze and thaw cycles and the analytical process. Short-term stability was tested after exposure of the plasma samples to ambient temperature for about 6 hours (the residence time of processing procedures of the sample). The freeze and thaw stability was evaluated after three freeze-thaw cycles at nominal −80°C. Sets of stability samples subjected to 3 freeze and thaw cycles at nominal –80°C were determined against freshly prepared calibration standards. Four measurements were made at each concentration of these QC samples. The storage stability in autosampler was determined by analysis the reconstituted QC samples after storing for about 24 hours at 8°C. Long-term stability was assessed after storage of the test samples at around −80°C for eight weeks. All stability tests were performed in freshly spiked calibration standards. The samples were considered stable in plasma at each concentration if the deviation from the active ingredient was considered stable in plasma if calculated relative standard deviation at each concentration of quality control sample was ±15%.

The stability of working solutions and stock solutions of RIS and its metabolite as well as internal standard was also determined at ambient room temperature for 12 hours and refrigerator for 15 days (below 10°C).

### 2.6. Pharmacokinetic Study

Eighteen male rats weighing 200 ± 10 g were divided into three groups, each group of 6 rats. The first group was administered the smallest dose (0.3 mg/kg) [27, 28], the second group was dosed with 1 mg/kg [29], and the dose for the third group was 6 mg/kg [30]. Animals which were kept in accordance with the recommendations of the “Guide for the Care and Use of Laboratory Animals” approved by the center were used in the study. Each group was placed individually in a cage under standard laboratory conditions (in 12 h light/dark cycle at 25°C ± 2°C provided with pellet diet and water). Blood samples were collected at each time point (before dose and at 0.5, 1, 2, 3, 5, 12, 24, and 48 hours after dose). The blood samples were collected in heparinized tubes. Collected samples were centrifuged at 4000 rpm and separated plasma samples were stored at −80°C until analysis. After analysis, different pharmacokinetics parameters of RIS and its metabolite were calculated for each rat. The pharmacokinetics parameters (*C*_max_, *t*_max_, AUC_0–48_, and AUC_0-inf_) were calculated. Noncompartmental analysis was applied to obtain the pharmacokinetic parameters using the WinNonlin® software, version 2.0 (Pharsight Corp., Mountain View, CA, USA). The calculated pharmacokinetics parameters for different dose levels were compared to find the dose proportional pharmacokinetics of risperidone and its metabolite.

## 3. Results

### 3.1. Method Development

Chromatographic conditions were optimized to achieve good sensitivity and symmetric peak shape for RIS, 9-RIS-OH, and IS, as well as a short run time. Several combinations with different ratios between solvents (acetonitrile; methanol) with format or acetate buffers to determine the optimum mobile phase for this separation were applied. Finally, a mobile phase consists of acetonitrile containing 0.1% formic acid and aqueous 5 mM ammonium acetate buffer containing 0.1% formic acid (80 : 20, v/v) at a flow rate 0.5 mL/min was employed on “Acquity UPLC BEH TM C_18_ column” (50 × 2.1 mm, i.d. 1.7 *μ*m). The strength and the ratio of the buffer are less than the other method reported by Bhatt et al. [25], so the column can achieve substantially longer lifetime by less exposure to buffer. After optimization, the retention times for RIS, 9-RIS-OH and IS were 1.01,0.95, and 1.04 min, respectively.

**Figure 2:**
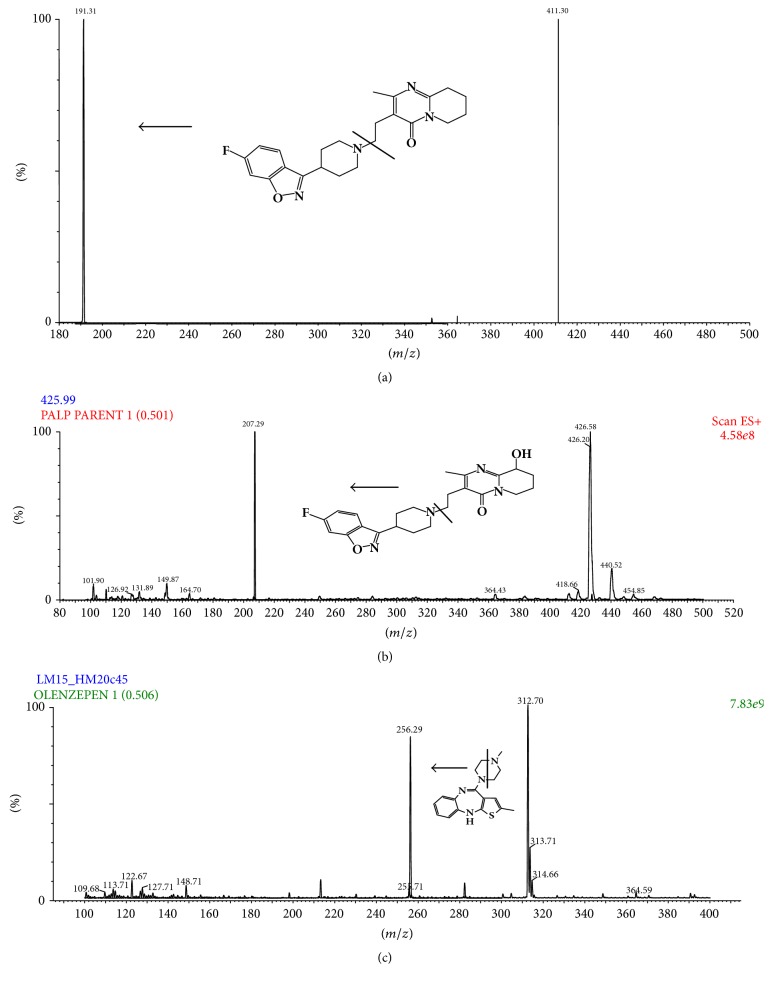
Mass spectra and the proposed patterns of fragmentation of (a) RIS, (b) 9-OH-RIS, and (c) OLA (IS).

MRM technique was chosen for the method development. To obtain a better response from the analytes electrospray ionization (ESI) was evaluated and it was found that the best signal was obtained by using the positive mode. The product ion mass spectra for RIS, 9-OH-RIS, and OLA yielded a high abundance of fragment ions of 191.31, 207.29, and 256.29, respectively Figure 2.

### 3.2. Sample Processing Optimization

Analytes and IS were extracted by protein precipitation in this study. Each of methanol and acetonitrile was used in precipitation. Finally, acetonitrile was found to be optimal, which can proceed with the highest recovery and produce a clean chromatogram for a blank plasma sample and for the analytes from plasma.

### 3.3. Method Performance and Validation

#### 3.3.1. Selectivity

**Figure 3:**
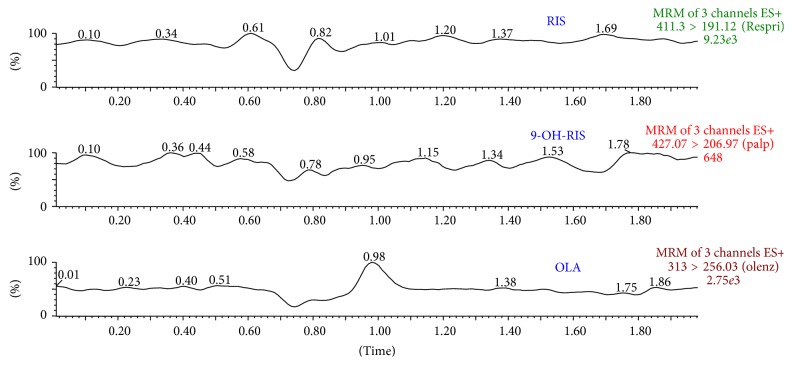
Representative MRM chromatograms of RIS, 9-OH-RIS, and IS (OLA) in blank plasma.

**Figure 4:**
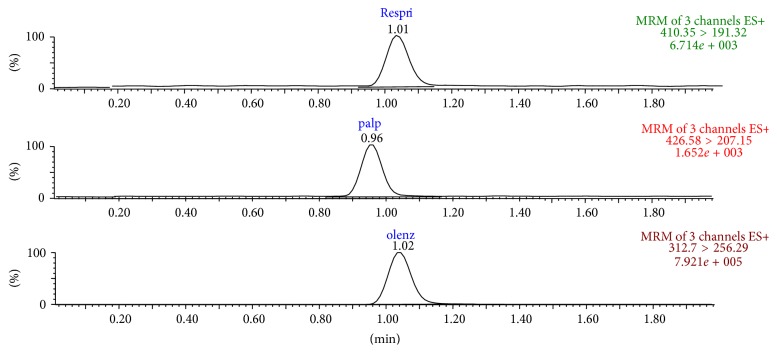
Representative MRM chromatograms of RIS, 9-OH-RIS, and IS (OLA) in spiked plasma.

**Figure 5:**
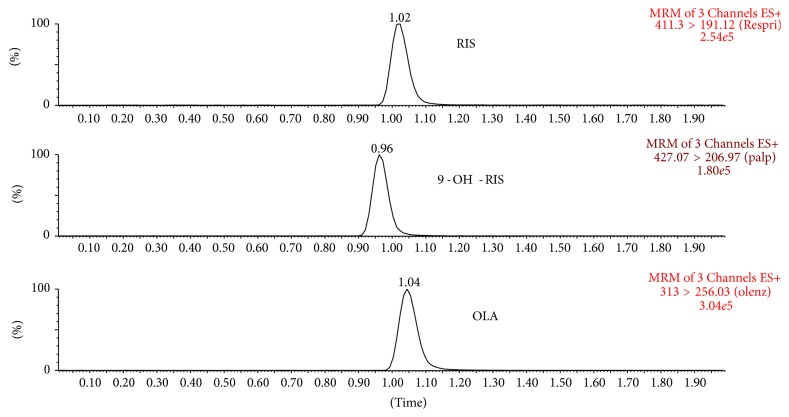
MRM chromatogram of plasma sample from rat at 1 h after administration of oral dosing of 1 mg/kg of RIS.

Method selectivity was evaluated by comparing the chromatograms of blank plasma (Figure 3) with* the* corresponding spiked with RIS, 9-OH-RIS, and OLA (IS) in plasma. No significant interference was appeared at the retention time of analytes and IS. The representative chromatogram of analyte and IS spiked at 100.0 ng/mL and after 1 hour of administration of RIS in rat is shown in Figures 4 and 5, respectively.

#### 3.3.2. Linearity and Sensitivity

**Figure 6:**
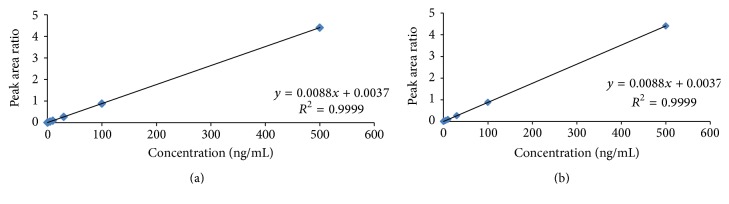
Standard calibration curve of RIS (a) and 9-OH-RIS (b).

The method linearity was estimated by the analysis of 8-point calibration curve for each of risperidone and its metabolite over a range of 0.2–500.0 ng/mL with the internal standard. The calibration curves were constructed by plotting area ratio (RIS/IS), (9-OH-RIS/IS) as a function of RIS and 9-OH-RIS concentrations. It was found to be linear from 0.2 to 500.0 ng/mL. The correlation coefficient (*r*) between concentration and peak area ratio was consistently greater than 0.999 during the course of validation (Figure 6).

#### 3.3.3. Precision and Accuracy

The intra- and interday accuracy and precision are shown in Tables 2 and 3. Accuracy of the intra- and interday for the determination of RIS and 9-OH-RIS was within 95.0–115.0%. The intra- and interday precision of the method, as determined from CV%, were less than 15% for both compounds.

#### 3.3.4. Recovery and Matrix Effects

Recovery of the present method was performed by comparing of the results obtained from the analysis of plasma spiked with three different concentrations of RIS and 9-OH-RIS (2.0, 20.0, and 400.0 ng/mL) with nonextracted samples. The recovery of this was 68.96%, 71.51%, and 69.56% for RIS and 70.29%, 65.59%, and 67.55% for 9-OH-RIS, respectively (Table 4). These results indicate that the efficiency of the recovery of this method is sufficient for the determination of RIS and 9-OH-RIS in plasma.

#### 3.3.5. Stability

The stabilities of RIS and 9-OH-RIS were evaluated at two concentrations of QC samples (2.0 and 400 ng/mL) during processing the analysis and sample storage (in-injector, bench-top, freeze/thaw and long-term stability tests). The stability results revealed that RIS and 9-OH-RIS spiked into plasma were stable for at least 6.0 hours at room temperature, for at least forty-eight hours in final extract under autosampler storage condition at 8°C, for sixty days at −80°C, and during three cycles of freeze-thaw (when stored at −80°C and thawed to room temperature) as shown in Table 5. These results revealed that there is no significant degradation of the RIS and 9-OH-RIS under analysis process or storage conditions evaluated. The stock solutions and working standard of RIS, 9-OH-RIS, and IS were also stable for two weeks at below 10°C (refrigerator temperature) and for 12 hours at room temperature. Therefore, RIS and 9-OH-RIS were deemed to be stable up to sixty days at −80°C in spiked plasma and up to two weeks in aqueous solution in refrigerator.

### 3.4. Dose-Dependent Pharmacokinetic Study in Rats

**Figure 7:**
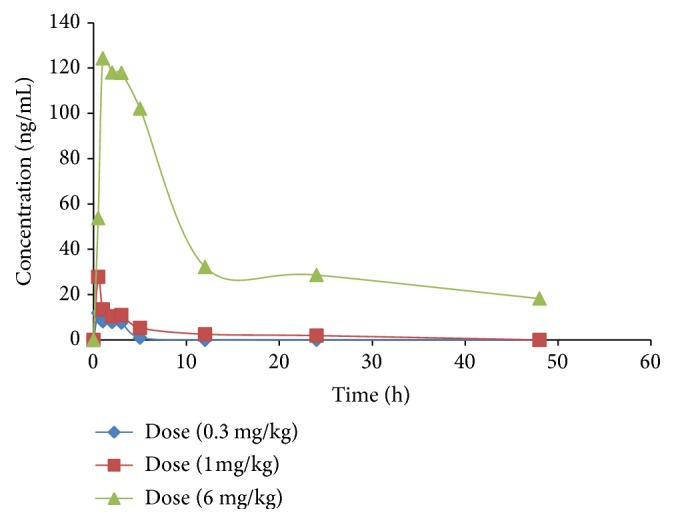
Mean plasma concentration-time profiles of RIS at three different doses.

**Figure 8:**
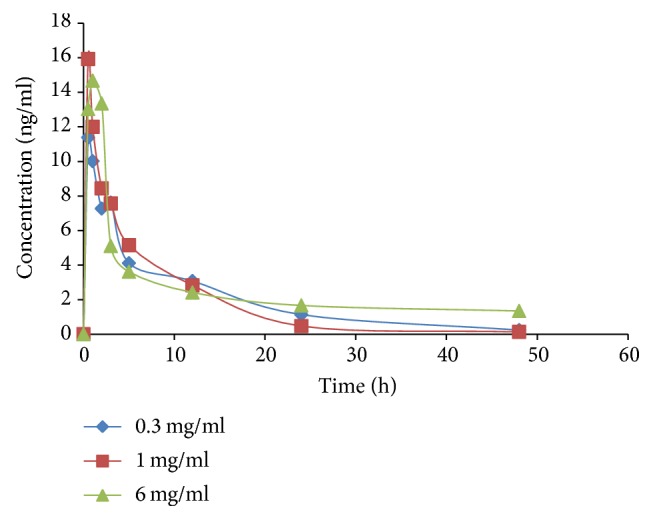
Mean plasma concentration-time profiles of 9-OH-RIS at three different doses.

The applicability of the validated method was demonstrated successfully in a pharmacokinetic study after administration of RIS at three different doses (0.3 mg/kg, 1.0 mg/kg, and 6.0 mg/kg) in rats. The mean plasma concentration-time profiles of RIS and its active metabolite at three doses are shown in Figures 7 and 8, respectively, whereas the major pharmacokinetic parameters of RIS and 9-OH-RIS are summarized in Table 6 for three different doses.

For risperidone, comparison of AUC_0–48_, AUC_0-inf_ values across dose levels demonstrated linearity in the dose response for the parent drug, risperidone, in low and middle doses. These parameters were not dose-dependent between dose levels of 1.0 and 6.0 mg/kg (6-fold difference between two doses). AUC_0–48_ increased from 101.81 ± 8.5 to 1914.83 ± 49.6 (18.8-fold) and from 126.01 ± 12.2 to 3094.49 ± 360.4 (24.5-fold) for AUC_0-inf_. Difference in dose-dependent pharmacokinetic studies was also observed in *C*_max_. The increase in the value of this parameter was less than proportional of exposure doses.

For risperidone metabolite, 9-hydroxyrisperidone, the values of AUC_0–48_ and AUC_0-inf_ increased by 1.2-fold and 0.95-fold, respectively, following increase in the doses from 0.30 to 1.0 mg/kg (3.3-fold). After increase of the dose from 1.0 to 6.0 mg/kg (6-fold), the value AUC_0–48_ and AUC_0-inf_ increased by 1.25-fold and 1.85-fold, respectively. *C*_max_ increased for about 2-fold following increasing doses from 0.3 to 1.0 mg/kg (3 fold), while insignificantly increased following raising doses from 1.0 to 6.0 mg/kg.

## 4. Discussion

Sensitive, fast, reliable, and economical, UPLC-MS/MS method was validated and developed for the determination of RIS and 9-OH-RIS in a small volume of plasma. The simplicity is one of the advantages of this method; it involved one step of protein precipitation using acetonitrile as precipitating agent. Comparing our method described here with that reported by Bhatt et al. [25], this method is characterized by a wide calibration range allowing its application in toxicokinetic and dose-dependent pharmacokinetic studies. Although our method recovery was lower than that reported by Bhatt et al., it is sufficient to detect terminal phase concentrations of the risperidone and its metabolite. The run time lasted only two minutes. Larger number of samples can be analyzed in a short time.

The proposed method could be practical and reliable for clinical, preclinical, toxicokinetic, and dose-dependent pharmacokinetic study of RIS and its active metabolite.

Dose response linearity in low and middle dose concerning *C*_max_ and AUC is compatible with the range of equivalent dose recommended for human. Disproportional and delayed maximum concentration at higher dose used only raises the possibility that risperidone may to some extent be absorbed by carrier-mediated transport mechanism that is saturated at this level.

AUC represents the total amount of drug (risperidone or 9-hydroxyrisperidone) involved in systemic exposure, which reflects the bioavailability and toxicity of the drug. At high dose (6.0 ng/kg), nonlinear increase in AUC was observed (6-fold increase in dose resulted in an increase by 18.8-fold AUC). Risperidone displays high protein binding. At the higher doses, protein binding sites are saturated and more risperidone remains free and available for metabolism and excretion [31]. The difference between risperidone and its metabolite in dose- response pharmacokinetics can be attributed to the differences in pharmacokinetics behavior of 9-hydroxyrisperidone. The absorption process of 9-hydroxyrisperidone (as a metabolite) is considered to be instantaneous and the drug is quickly distributed via the circulatory system [32].

## 5. Conclusion

Highly simple and reproducible UPLC-MS/MS method was developed and validated for the simultaneous determination of RIS and 9-OH-RIS in a small volume of plasma. The method is fast rather than extraction method as it needs only one-step protein precipitation in less than 2 minutes. The proposed method is successfully applied to dose proportional pharmacokinetic study of RIS and 9-OH-RIS in rats at a wide range of dose.

## Figures and Tables

**Table 1:** Mass optimization parameter for RIS, 9-OH-RIS, and OLA.

Parameters	RIS	9-OH-RIS	IS
Cone voltage (V)	45	45	40
Collision energy (eV)	30	25	20
The collision gas (argon) flow rate (ml/min)	0.1	0.1	0.1
Nitrogen flow rate	600 L/h	600 L/h	600 L/h

**Table 2:** Intra- and interday precision and accuracy of RIS in rat plasma.

Theoretical conc. (ng/mL)	Intraday			Interday		
	Measured conc. (ng/mL) (mean ± SD)	Precision (CV%)	Accuracy (%)	Measured conc. (ng/mL) (mean ± SD)	Precision (CV%)	Accuracy (%)
0.2	0.21 ± 0.004	7.30	105.00	0.23 ± 0.01	8.33	115.0
2	1.99 ± 0.05	3.77	99.50	1.97 ± 0.13	5.69	98.50
20	19.19 ± 0.01	5.88	95.95	20.1 ± 2.70	3.99	100.50
400	395 ± .020	7.57	98.75	398 ± 1.35	7.15	99.50

**Table 3:** Intra- and interday precision and accuracy of 9-OH-RIS in rat plasma.

Theoretical conc. (ng/mL)	Intraday			Interday		
	Measured conc. (ng/mL) (mean ± SD)	Precision (CV%)	Accuracy (%)	Measured conc. (ng/mL) (mean ± SD)	Precision (CV%)	Accuracy (%)
0.2	0.19 ± 0.01	9.11	95.00	0.22 ± 0.08	5.11	110.00
2	2.1 ± 0.02	3.77	105.00	1.95 ± 0.39	8.45	97.50
20	19.39 ± 1.51	5.88	96.95	20.1 ± 1.27	7.98	100.50
400	392 ± 2.35	7.57	98.00	395 ± 0.07	4.35	98.75

**Table 4:** Recovery data of RIS 9-OH-RIS (3 QC samples) and olanzapine.

Compound	Nominal conc. (ng/mL)	Recovery (% )
RIS (analyte)	2.0	68.96
	20.0	71.51
	400.0	69.56

9-OH-RIS (metabolite)	2.0	70.29
	20.0	65.99
	400.0	67.55

Olanzapine (IS)	1200.0	68.90

**Table 5:** Stability of RIS and 9-OH-RIS in rat plasma.

Stability	Nominal conc. (ng/mL)	RIS			9-OH-RIS		
		Measured conc. (ng/mL) (mean ± SD)	Precision (CV%)	Accuracy (%)	Measured conc.(ng/mL) (mean ± SD)	Precision (CV%)	Accuracy (%)
Bench top	2	2.19 ± 0.07	8.18	109.50	2.15 ± 0.03	6.76	107.5
(6 h)	400	396 ± 1.59	5.85	99.00	397 ± 0.05	8.54	99.25
Freeze thaw	2	2.34 ± 0.07	6.58	117.00	1.97 ± 1.08	7.98	98.5
(3 cycles)	400	394 ± 20.12	5.51	98.50	390 ± 0.07	5.90	97.5
In injector	2	1.98 ± 1.07	4.14	99.00	1.89 ± 2.35	7.95	94.5
(48 h)	400	377 ± 15.00	7.18	94.25	392 ± 12	7.12	98.0
60 days	2	1.88 ± 0.08	3.95	94.00	1.79 ± 3.31	6.55	89.5
at −80°C	400	390 ± 12.15	4.59	97.50	393 ± 10.21	4.24	98.25

**Table 6:** Mean pharmacokinetic parameters (mean ± SD) of RIS and 9-OH-RIS following administration of different doses of RIS to rats.

Dose (mg/kg)	*C*_max_ (ng/ml)		*t*_max_ (h)^*∗*^		AUC_0–48_ (ng·h/ml)		AUC_0-inf_ (ng·h/ml)		*t*_1/2_ (h)		MRT (h)		*K*_*el*_ (h)	
	RIS	9-OH-RIS	RIS	9-OH-RIS	RIS	9-OH-RIS	RIS	9-OH-RIS	RIS	9-OH-RIS	RIS	9-OH-RIS	RIS	9-OH-RIS
0.3	11.96 ± 2.4	7.95 ± 1.6	0.5	1.0	32.52 ± 0.5	79.9 ± 1.6	38.15 ± 2.2	102.8 ± 43.7	1.21 ± 0.3	17.9 ± 17.04	2.63 ± 0.4	0.075 ± 0.03	0.59 ± 0.2	12.9 ± 10.6
1.0	27.37 ± 3.5	15.92 ± 4.05	0.5	0.5	101.81 ± 8.5	95.7 ± 28.1	126.01 ± 12.2	97.2 ± 28.1	9.53 ± 2.7	8.1 ± 1.8	13.09 ± 3.9	0.11 ± 0.04	0.08 ± 0.01	6.5 ± 1.8
6.0	125.09 ± 2.4	14.6 ± 0.46	1.0	1.0	1914.83 ± 49.6	120.34 ± 24.6	3094.49 ± 360.4	179.96 ± 9.2	43.62 ± 7.7	32.4 ± 3.09	51.88 ± 9.8	0.02 ± 0.002	0.02 ± 0.003	25.16 ± 2.3

^*∗*^Median for *t*_max…_
